# Intelligent Informatics in Biomedicine

**DOI:** 10.1155/2013/185839

**Published:** 2013-04-22

**Authors:** Hao-Teng Chang, Raffaele A. Calogero, Sorin Draghici, Oliver Ray, Tun-Wen Pai

**Affiliations:** ^1^Graduate Institute of Basic Medical Science, College of Medicine, China Medical University, Taichung, Taiwan; ^2^Department of Clinical and Biological Sciences, Torino University, Torino, Italy; ^3^Department of Computer Science, Wayne State University, Detroit, MI 48202, USA; ^4^Department of Computer Science, University of Bristol, Bristol BS8 1UB, UK; ^5^Department of Computer Science and Engineering, National Taiwan Ocean University, No. 2 Peining Road, Keelung 20224, Taiwan; ^6^Center for Marine Bioenvironment and Biotechnology, National Taiwan Ocean University, No. 2 Peining Road, Keelung 20224, Taiwan

In the postgenomic era, hundreds of thousands of biological datasets including genetic information of various species, gene expression profiles, metabolomes, proteomes, and even molecular imaging are published in public domains. One, maybe the most, important reason to conduct various genome projects is to translate useful relevant information to biomedical research and finally to clinical applications. From bench to bed, bioinformatics researches have presented strong and powerful potential to accelerate the analyses of comprehensive and complicated datasets. To establish a forum for gathering scientists from multidisciplinary fields such as biology, medicine, computer science, statistics, and informatics, Dr. Hui-Huang Hsu, Dr. Tun-Wen Pai, Dr. Oliver Ray, and Dr. Hao-Teng Chang are continuously involved in organizing International Workshop on Intelligent Informatics in Biology and Medicine (IIBM) starting from 2008, which is used to be held in conjugation with International Conference on Complex, Intelligent, and Software Intensive Systems (CISIS). 

In these years, IIBM successfully brings together computer scientists, biologists, statisticians, and medical doctors to present and discuss current topics on intelligent informatics in biology and medicine. Although the complexity and volume of experimental data from next generation sequencing and mass spectrometry technologies increase dramatically, many various sophisticated computational techniques have been designed and developed. These methodologies are able to support new detection techniques that are developed to improve the quality of healthcare and medicine. Advances in information technologies, indeed, facilitate and accelerate research from basic to clinical investigations in terms of translational medicine.

To record the ideas of talents and gather more contributions to these fields, this special issue was launched and supported by this journal. This special issue focuses on the challenges and solutions for information process with an emphasis on forthcoming high throughput technologies and biomedicine systems, which will provide opportunities for academics and industrial professionals to discuss the latest issues and progresses in the area of biomedicine. This special issue contains 9 papers which were selected from 24 submissions. These papers address the development and application of data-analytical methods, algorithm development, mathematical modeling, and computational simulation techniques to the biomedicine applications. 

In “*Time series expression analyses using RNA-seq: a statistical approach*,” S. Oh et al. apply three real datasets and simulation studies to demonstrate the utility of statistical evolutionary trajectory index, autoregressive time-lagged regression, and hidden Markov model approaches for RNA-seq datasets in temporal version.

In “*Gene entropy-fractal dimension informatics with application to mouse-human translational medicine*,” T. Holden et al. compare the Shannon entropy and fractal dimension of some DNA sequences computed from different mammalian species. The obtained values were plotted in a 2D map, and the distance between points on these maps for corresponding mRNA sequences in different species is used to study evolutionary topics. 

In “*State-of-the-art fusion-finder algorithms sensitivity and specificity*,” M. Carrara et al. utilize seven existing state-of-arts gene fusion detection tools. Their strategy is to simulate some gold-standard data and then compare sensitivity and specificity of the detection tools. The experimental results obtained using synthetic and real datasets suggest that synthetic datasets encompassing fusion events may not fully catch the complexity of RNA-seq experiments, and most fusion detection tools are still limited in sensitivity or specificity. 

In “*On the structural context and identification of enzyme catalytic residues*,” Y.-T. Chien and S.-W. Huang analyze structural context of catalytic residues based on theoretical and experimental structure flexibility. The results have shown that catalytic residues possess distinct structural features and contexts, and their neighboring residues within specific range are usually structurally rigid than those of noncatalytic residues.

In “*Simpute: an efficient solution for dense genotypic data*,” Y.-J. Lin et al. compare the imputation performance among six various bioinformatics tools with data generated by randomly masking the genotype data from the International HapMap Phase III Project. They also propose a novel algorithm, Simpute, which is suitable and efficient for regular screening of the large-scale SNP genotyping in general.

 In “*In silico prediction and in vitro characterization of multifunctional human RNase3*,” P.-C. Lien et al. apply computational approaches for unique peptide extraction and perform *in vitro* activity assays for discovering important peptides in human ribonuclease 3 (hRNase3), HBP*rnase3*. They also identify multiple biological features of this unique peptide in glycan binding, cellular binding, and lipid binding, which are also characteristic features of hRNase 3. Their results demonstrate molecular evolution of sequence, structure, and function in human ribonuclease A (hRNaseA) superfamily members.

In “*Using nanoinformatics methods for automatically identifying relevant nanotoxicology entities from the literature*,” M. García-Remesal et al. present a nanoinfomatics approach based on NER techniques for automatically identifying relevant nanotoxicology entities in scientific papers. The proof of concept can be expanded to stimulate further developments that could assist researchers in managing data, information, and knowledge at nanolevel and accelerating research in nanomedicine.

In “*On the difference in quality between current heuristic and optimal solutions to the protein structure alignment problem*,” M. Arriagada and A. Poleksic utilize an approximation algorithm for protein structure matching to demonstrate that a deep search of the protein superposition space leads to increased alignment accuracy with respect to many well-established measures of alignment quality. The topic of protein structure alignment is still one of the most important problems in computational biology. 

In “*Cancer vaccines: state of the art of the computational modeling approaches*,” F. Pappalardo et al. introduce the new field of computational modeling of cancer vaccines which are a real application of the extensive knowledge of immunology to the field of oncology.

The papers in this special issue, representing a broad spectrum of computational approaches and areas of investigation, provide useful message of intelligent informatics for biomedical and biomedicine applications. This unique and informative collection of papers on bioinformatics highlights the direction of related studies. This special issue illustrates the importance that computational biology always plays the primary key step for biologists and medical doctors to be able to access large amounts of biological data.

